# Pain, function, and acute neuropathic pain up to one month after surgery: a registry-based analysis

**DOI:** 10.1097/PR9.0000000000001456

**Published:** 2026-06-17

**Authors:** Ana Lilia Garduño-López, Philipp Baumbach, Victor Manuel Acosta-Nava, Frida Fernanda Verdugo-Velázquez, Gabriela Josefina Vidaña-Martínez, Eldeli Molina-Niño, Luis Felipe Cuellar-Gúzman, Belén Aurora García-Herrera, Dulce María Rascón-Martínez, Gabriela Islas-Lagunas, Juan de la Cruz Pineda-Pérez, J. Jesús Cano-García, Mariana Calderon-Vidal, Jorge Jimenez-Tornero, Oscar López-Hernández, Elizabeth Villegas-Sotelo, Monica Dominguez-Cid, Maria Esther Flores-Villanueva, Winfried Meissner, Marcus Komann, Christin Arnold, Claudia Weinmann, Ulrike M. Stamer, Ruth Zaslansky

**Affiliations:** a Department of Anesthesiology, Instituto Nacional de Ciencias Medicas y Nutrición Salvador, Zubirán, Mexico; b Department of Anesthesiology and Intensive Care Medicine, Jena University Hospital, Friedrich Schiller University, Jena University Hospital, Jena, Germany; c Department of Anesthesiology, Hospital Central Dr. Ignacio Morones Prieto, San Luis Potosí, Mexico; d Department of Anesthesiology, Hospital Central Morones Prieto, San Luis Potosí, Mexico; e Department of Anesthesiology, Instituto Nacional de Cancerología, Mexico City, Mexico; f Department of Anesthesiology, Centro Médico Nacional Siglo XXI-IMSS, Mexico City, Mexico; g Department of Anesthesiology, Instituto Nacional de Enfermedades Respiratorias Cosio Villegas, Mexico City, Mexico; h Department of Anesthesiology, Hospital Aranda de La Parra, Gto, Mexico; i Department of Anesthesiology, Hospital Fundación Medica Sur, Mexico City, Mexico; j Department of Anesthesiology, Hospital San Javier, Guadalajara, Mexico; k Department of Anesthesiology, Hospital General Dr Rubén Leñero, Mexico City, Mexico; l Department of Anesthesiology, Hospital Angeles Puebla, Pue, Mexico; m Department of Anesthesiology, IMSS Hospital General Regional Núm. 2 de Villacoapa, CDMX, Mexico; n Department of Anesthesiology and Pain Medicine, Inselspital Bern University Hospital, Bern, Switzerland

**Keywords:** Postoperative pain, Surgery, Acute neuropathic pain, Recovery, Risk factors, Subacute pain

## Abstract

**Introduction:**

Knowledge of how patients experience pain and patterns of resolution beyond the first days after surgery is limited. Acute neuropathic pain (ANeuP) following surgery is rarely assessed.

**Objectives:**

We aimed to characterize pain intensity, pain-related functional impairment, ANeuP, and to describe patterns of resolution in a large cohort undergoing diverse surgical procedures up to 1 month postoperatively, the subacute period.

**Methods:**

This was an observational, multicentre study. Outcomes were assessed on postoperative days (PODs) 1/7/30 using the PAIN OUT registry methodology. The primary endpoint was the change in pain-related interference between POD7 and POD30. Patient subgroups were identified using cluster analysis.

**Results:**

A cohort of 1,153 patients (median age 49; 67% female) was enrolled. By POD30, pain-related functional interference decreased (*P* < 0.001, moderate-to-large effect). The cluster analysis identified 3 subgroups: Minimal-pain resolution (16% of the cohort), Slow-pain resolution (30.5%), and Fast-pain resolution (53.5%). By POD30, patients in the Slow- and Fast-pain resolution clusters had largely recovered, differing in recovery rate. Those in the Minimal-pain resolution cluster showed limited improvement from POD1. By POD30, these patients still experienced mild–moderate pain and interference, with 40% screening positive for ANeuP and 24% taking opioids. Risk factors included chronic preexisting pain and thoracic or orthopaedic surgery.

**Conclusion:**

Although the majority of patients in the cohort largely recovered in terms of pain and interference by POD30, 16% belonged to the Minimal-pain resolution cluster. These patients may benefit from proactive identification and management by anaesthetists, surgeons, and practitioners in the community before surgery or in the early weeks after surgery.

## Abstract 2

Supplemental Digital Content is Available in the Text.

Among 1,153 surgical patients, 84% recovered by 1 month, and 16% reported pain and interference. Chronic pain history and thoracic/orthopaedic surgery predicted risk, supporting early detection.

## 1. Introduction

Many studies have evaluated pain-related patient-reported outcomes (PROs) and treatments after surgery, often focusing on the first postoperative day.^8,35^ Knowledge of patients' pain experiences and recovery patterns beyond the initial 24 hours remains limited.^12^ Acute pain is typically defined as lasting up to 7 days after an inciting event.^15^ Whereas, according to ICD-11, chronic postsurgical (CPSP) or chronic posttraumatic pain is pain that develops or increases in intensity after a surgical procedure or tissue injury, persisting beyond the healing process at least for 3 months after the initiating event.^24^ The intermediate period, termed the “subacute” postoperative pain, represents a poorly defined period in which acute pain may transition to chronic pain. This phase, spanning from approximately 72 hours to 12 weeks after surgery, has been described as a “gray zone.”^5,16^ It is accepted that during this period, most patients regain functional capacity, although recovery is not universal.^29^ During this period, pain management may be particularly challenging as once patients are discharged from the hospital, they assume responsibility for their own medications, and accountability for treatment is often ambiguous, falling between surgeon and general practitioner.^37^ Adolescents who developed CPSP reported significantly more days of severe pain and functional interference during the subacute period compared with those who did not progress to CPSP.^21^ However, despite its clinical importance, relatively little is known about the mechanisms underlying pain resolution and functional recovery during the subacute phase.^16^ At present, the term “subacute” remains descriptive until more precise characterization in terms of clinical features, diagnostic criteria, prognosis, and treatment becomes possible.^15^

Another area of uncertainty concerns the acute manifestations of postsurgical nerve injury. Acute neuropathic pain (ANeuP), defined as pain occurring within 30 days of peripheral nerve injury (eg, during surgery), distributed in a neuroanatomically plausible manner, accompanied by at least 1 sensory change, and sometimes functional impairment, remains underexplored. Data on its prevalence, clinical characteristics, and optimal management are limited.^10^

### 1.1. Study objectives

The objectives of this study were to characterize pain intensity, pain-related functional interference, and ANeuP in a large cohort of patients undergoing diverse surgical procedures in the clinical routine during the acute and subacute postoperative phases.

We hypothesized that, although pain intensity and pain-related functional interference would improve over time across the cohort, recovery in terms of resolution of pain and related outcomes would not be uniform. Instead, we anticipated that patients would cluster into distinct subgroups based on different patterns of pain intensity, functional recovery, and ANeuP. Such information may enable anaesthetists, surgeons, and community practitioners to identify patients at risk of impaired recovery and intervene proactively, either preoperatively or during the early postoperative period.^14^

## 2. Methods

### 2.1. Study design, setting, and participants

This was an observational, cohort study in which adult patients undergoing diverse surgical procedures were studied prospectively at 3 time points, up to 1 month after surgery, in 11 tertiary hospitals in Mexico. The study was coordinated by 2 local physicians (A. G. and V. A.) and a Principal Investigator in each hospital. The ethics committee of each hospital approved the study protocol (see supplemental digital content, Material 1, http://links.lww.com/PR9/A415). The trial methodology and oversight were provided by PAIN OUT, an international perioperative pain registry (www.pain-out.eu). The trial was registered in Clinical Trials Gov under NCT05315596. The STROBE guidelines guided the preparation of this manuscript.^39^

Patients met the following inclusion criteria: (1) ≥18 years; (2) on the first postoperative day (POD); and (3) consented to participate in the survey assessing pain-related outcomes at 3 time points, POD1, POD7, and POD30. Patients completed the assessments by filling out questionnaires independently, using a link sent to their phone, or by interview conducted by a surveyor. Anaesthesia residents, not involved in patient care, served as study surveyors. They entered the findings into a password-protected web-based portal. Each dataset was assigned a unique, anonymous code, unconnected to the patient's name or the original medical record.

### 2.2. Variables for analysis of outcomes and processes

#### 2.2.1. Patient characteristics, anaesthesia, and surgical information

Information on the patient's age, sex, type of surgery (based on International Classification of Diseases procedure codes [ICD9]), anaesthetic technique, analgesics administered perioperatively, duration of surgery, and time between the end of surgery and when the patient survey was carried out was extracted from the patients' medical charts. The PI in each hospital selected surgical disciplines for follow-up.

#### 2.2.2. Pain-related patient-reported outcomes

Pain-related PROs were assessed at 3 time points.

On POD1, patients completed the validated International Pain Outcomes Questionnaire,^22^ which evaluates the following aspects of the pain experience:

(1) Pain intensity: worst and least pain, percentage of time in severe pain.
(2) Pain-related Interference: Impact of pain on activities in bed, sleep, emotions, anxiety, helplessness.
(3) Side effects: nausea, drowsiness, itch, dizziness.
(4) Pain for at least 3 months before surgery (=chronic pain).

Most PROs were measured using a 0 to 10 numerical rating scale (NRS), where “0” represents “no pain/interference,” and “10” signifies “worst pain/complete interference.” Time in severe pain was rated as a percentage, 0% to 100%, in 10% increments.

The Pain Composite Score-Total (PCS) was calculated by averaging all continuous items from the pain intensity, pain interference, and side effects domains. Subcomposite scores were also generated separately for each domain: intensity, interference (physical and emotional), and side effects.^41^

On PODs, 7 and 30 patients completed the:

(1) The Brief Pain Inventory Short Form (BPI),^2^ evaluates 2 key domains of pain: intensity (worst, least, average, and current pain) and pain-related interference with daily life. Interference is further divided into a *physical* subdomain (general activity, walking ability, and work) and an *affective* subdomain (mood, enjoyment of life, and relationships with others). Each item is rated on a 0 to 10 NRS. The *Pain Interference Total Score (PITS)* was derived by averaging the 7 interference items, creating a composite measure assessing pain-related functional impairment. Patients were categorized into groups based on their PITS: no interference (PITS = 0), mild (PITS = 1–2), moderate (PITS = 2–5), and severe interference (PITS > 5).^26^ Mean scores for the 2 functional subdomains—physical and affective interference—were also calculated. The *Pain Severity Score (PSVS)* was determined by averaging the 4 pain intensity variables. Sleep was analysed separately, as its inclusion did not enhance the psychometric properties of the BPI interference scale.
(2) Douleur Neuropathique 2 (DN2, interview version of DN4) to screen for symptoms of neuropathic pain in relation to the surgical incision using a yes/no format. Neuropathic pain is possible if ≥ 3/7 symptoms are selected.^1^

### 2.3. Study endpoints

The primary endpoint was the difference in pain *interference* scores (PITSs) between POD7 and 1 month after surgery (POD30). We also compared the number of patients with no, mild, moderate, and severe PITS between POD7 and POD30.

Secondary endpoints included differences on POD7 and POD30 for (1) Pain *Severity* Score (PSVS), (2) the proportion of patients with a positive screening for acute neuropathic pain, (3) the proportion and types of analgesics taken (any, nonopioid, opioid, co-analgesics/adjuvants [ie, anticonvulsants and antidepressants]).

Additional endpoints consisted of identifying patient subgroups (clusters) related to pain severity and pain interference on POD1, POD7, and POD30.

### 2.4. Sample size

The project was designed primarily as a quality improvement measure in individual wards.

Clinicians were asked to collect at least 60 full datasets for each ward, a figure regarded as adequate to represent the care provided in each ward and to provide sufficient patient numbers for the secondary analyses. No statistical sample size estimation was conducted for the current analysis.

### 2.5. Statistical analysis

#### 2.5.1. Descriptive statistics

Categorical variables are reported as absolute (n) and relative (%) values, and metric variables as median and interquartile range (IQR). For POD1, datasets were valid if inclusion criteria and Pain Composite Score variables were met; for POD7 and POD30, if PITS variables were available. A dataset was excluded if a patient could not be reached after 3 phone call attempts.

#### 2.5.2. Clustering

We applied k-means clustering to group patients by pain intensity and interference at POD1 (IPO-Q: Pain Composite Scores) and by pain severity and interference at POD7 and POD30 (BPI: PSVS and PITS). All variables were z-standardized before clustering. The optimal number of clusters (k) was evaluated using the *fviz_nbclust* function of the factoextra package (version 1.0.7), and the final cluster analysis was performed using the *clusterboot* function of the fpc package (version 2.2–10; details in supplemental digital content, Material 2, http://links.lww.com/PR9/A415).

#### 2.5.3. Inferential statistics

Differences in categorical and metric variables between POD7 and POD30 (paired analyses) were tested using the McNemar-Bowker test and the Wilcoxon signed-rank test, respectively. In addition, we reported the corresponding effect sizes Cohen G (categorical variables; absolute values ≥0.05 small, ≥0.15 moderate, ≥0.25 large^4^) or r (metric variables; absolute values ≥0.1 small, ≥0.3 moderate, ≥0.5 large^7^).

Differences between subgroups (cluster comparisons) in categorical and metric variables were tested using the χ^2^ test, Kruskal-Wallis test, and Mann-Whitney *U* test. In addition, we reported the corresponding effect sizes, Cohen's ω (categorical variables; absolute values ≥0.1 small, ≥0.3 moderate, ≥0.5 large^4^), eta squared (metric variables in >2 groups; absolute values ≥0.01 small, ≥0.06 moderate, ≥0.14 large^3,33^), or r (metric variables in 2 groups; absolute values ≥0.1 small, ≥0.3 moderate, ≥0.5 large^7^).

Logistic regression identified potential risk factors for worse outcomes, using membership in the worst-outcome subgroup as the dependent variable and demographic and process variables (Table 1) as predictors. We first built a full model, then applied backward selection based on the Akaike Information Criterion (AIC) using the *stepAIC* function (MASS package, version 7.3–58.2).

**Table 1:** Patient characteristics and process variables for the complete cohort.

	Total N = 1,153
Age (y), median (IQR)	49 (35–63), n = 1,150
Duration of surgery (h), median (IQR)	2.4 (1.7–3.6), n = 1,134
Time to survey after surgery (h), median (IQR)	24.3 (20.1–26.2), n = 1,106
Sex (female), n (%)	770/1,142 (67.4%)
Preexisting chronic pain (yes), n (%)	335/1,150 (29.1%)
Opioids taken before admission	67/1142 (5.9%)
Surgical discipline (%)	
General surgery	438 (38%)
Obstetrics/gynaecology	289 (25.1%)
Thoracic surgery	187 (16.2%)
Traumatology/orthopaedics	134 (11.6%)
Urology	105 (9.1%)
Intraoperative anaesthesia, n (%)	
Regional (only)	291/1,141 (25.5%)
General (only)	414/1,141 (36.3%)
Regional and general	436/1,141 (38.2%)
Intraoperative wound infiltration (yes), n (%)	157/1,123 (14.0%)
Opioid: intraoperative, n (%)	
None	55 (4.8%)
Systemic (only)	876 (76.0%)
Regional (only)	131 (11.4%)
Systemic and regional	91 (7.9%)
Opioid: recovery room (any), n (%)	359 (31.1%)
Opioid: ward (any), n (%)	669 (58.0%)
Nonopioid: analgesics intraoperative (any), n (%)	890 (77.2%)
Nonopioid: analgesics recovery room (any), n (%)	261 (22.6%)
Nonopioid: analgesics ward (any), n (%)	1,026 (89.0%)

IQR, interquartile range; in cases with missing data, the number of patients included in the analysis is specified.

The authors approved the analysis plan before commencing the analysis. For the analysis, we used R (version 4.1.0, R Foundation for Statistical Computing, Vienna, Austria) and RStudio (version 1.4.1717, Boston, MA^23^). Statistical significance was set at *P* < 0.05.

## 3. Results

Between January 2022 and June 2023, surveyors approached 2,185 patients from 25 wards in 14 hospitals. On POD1, 1,992 patients fulfilled the inclusion criteria (data were incomplete for n = 193 patients). On POD7, the cohort included data from 1,294 patients (n = 602 could not be reached, and data were incomplete for n = 96). On POD30, the cohort for the analysis included data from 1,153 patients (n = 91 could not be reached, and data were incomplete for n = 50). See flow chart in supplemental digital content, Material 3 (http://links.lww.com/PR9/A415).

### 3.1. Patient characteristics and surgical data

Table 1 summarizes the descriptive statistics of the demographic and surgical variables across the cohort. Patients were 49 (35–63) years old, 67.4% were female, and 29% reported experiencing chronic pain before surgery. The cohort consisted of patients undergoing procedures related to 5 surgical disciplines: general surgery, obstetrics and gynaecology, thoracic surgery, traumatology, orthopaedics, and urology. The most frequent surgical procedures are listed in supplemental digital content, Material 4 (http://links.lww.com/PR9/A415). Surgery duration was 2.4 (1.7–3.6) hours, and patients were approached to join the survey 24.3 (20.1–26.2) hours after surgery.

### 3.2. Pain-related patient-reported outcomes

#### 3.2.1. Primary endpoint

Between POD7 and POD30, pain interference (BPI: PITS) decreased significantly (*P* < 0.001) with a moderate-to-large effect size (Table 2). The proportion of patients with no, mild, moderate, or severe pain interference differed significantly between the 2 time points (*P* < 0.001, large effect size; Table 2).

**Table 2:** Summary of pain interference and severity on postoperative days 7 and 30.

	Day 7 N = 1,153	Day 30 N = 1,153	*P*	ES
Pain interference				
PITS: total (0–10 NRS), median (IQR)	1.1 (0.0–3.3)	0.3 (0.0–1.6)	**<0.001**	0.48 (medium)
PITS: total (categorized), n (%)			**<0.001**	0.26 (large)
None (0/10 NRS)	308 (26.7%)	501 (43.5%)		
Mild (0–2/10 NRS)	425 (36.9%)	410 (35.6%)		
Moderate (>2-5/10 NRS)	282 (24.5%)	171 (14.8%)		
Severe (>5/10 NRS)	138 (12.0%)	71 (6.2%)		
PITS: physical (0–10 NRS), median (IQR)	1.7 (0.0–4.3)	0.3 (0.0–2.0)	**<0.001**	0.5 (large)
PITS: affective (0–10 NRS), median (IQR)	0.3 (0.0–2.3)	0.0 (0.0–1.0)	**<0.001**	0.41 (medium)
Sleep (0–10 NRS), median (IQR)	0.0 (0.0–3.0)	0.0 (0.0–1.0)	**<0.001**	0.4 (medium)
Pain severity				
PSVS: total (0–10 NRS), median (IQR)	1.5 (0.5–3.3)	0.8 (0.0–2.0)	**<0.001**	0.49 (medium)
Average pain (0–10 NRS), median (IQR)	2.0 (0.0–3.0)	0.0 (0.0–2.0)	**<0.001**	0.49 (medium)
Worst pain (0–10 NRS), median (IQR)	3.0 (1.0–5.0)	1.0 (0.0–3.0)	**<0.001**	0.49 (medium)
DN2 positive, n (%)	231 (20%)	185 (16%)	**0.003**	0.1 (small)
Analgesics, n (%)				
Any	938/1,146 (81.8%)	435/1,146 (38.0%)	**<0.001**	0.44 (large)
Nonopioid analgesics	878/1,146 (76.6%)	392/1,146 (34.2%)	**<0.001**	0.41 (large)
Opioid	333/1,146 (29.1%)	120/1,146 (10.5%)	**<0.001**	0.38 (large)
Co-analgesics/adjuvants	69/1,146 (6.0%)	55/1,146 (4.8%)	0.12	0.1

In cases with missing data, the number of patients included in the analysis is specified.

DN2, *Douleur Neuropathique* 2; ES, effect sizes vary by the type of variable assessed, Cohen's G effect size (categorical variables; absolute values ≥0.05 small, ≥0.15 medium, ≥0.25 large) or r (metric variables; absolute values ≥0.1 small, ≥0.3 medium, ≥0.5 large); *P*: *P* value of the McNemar-Bowker tests (categorical variables) or Wilcoxon signed-rank tests (metric variables), *P* values <0.05 are printed in bold; PITS, Pain Interference Scale; PSVS, Pain Severity Scale. Co-analgesics: anticonvulsants/antidepressants.

#### 3.2.2. Secondary endpoints

Pain severity (BPI: PSVS), the number and proportion of patients with a positive DN2, and those taking analgesics decreased significantly between the 2 time points (all *P* < 0.01), with varying effect sizes. The use of co-analgesics/adjuvants did not vary between the 2 time points (see Table 2).

### 3.3. Additional endpoints (trajectory clusters)

The indices for obtaining the optimal number of clusters (k) indicated a 2- to 3-cluster solution (see supplemental digital content, Material 2, http://links.lww.com/PR9/A415). For the main analysis, we focused on a 3-cluster solution. The results for the 2-cluster solution are presented in supplemental digital content, Material 5, http://links.lww.com/PR9/A415.

### 3.3.1. Patient-reported outcomes and medications for pain

**Figure 1.**
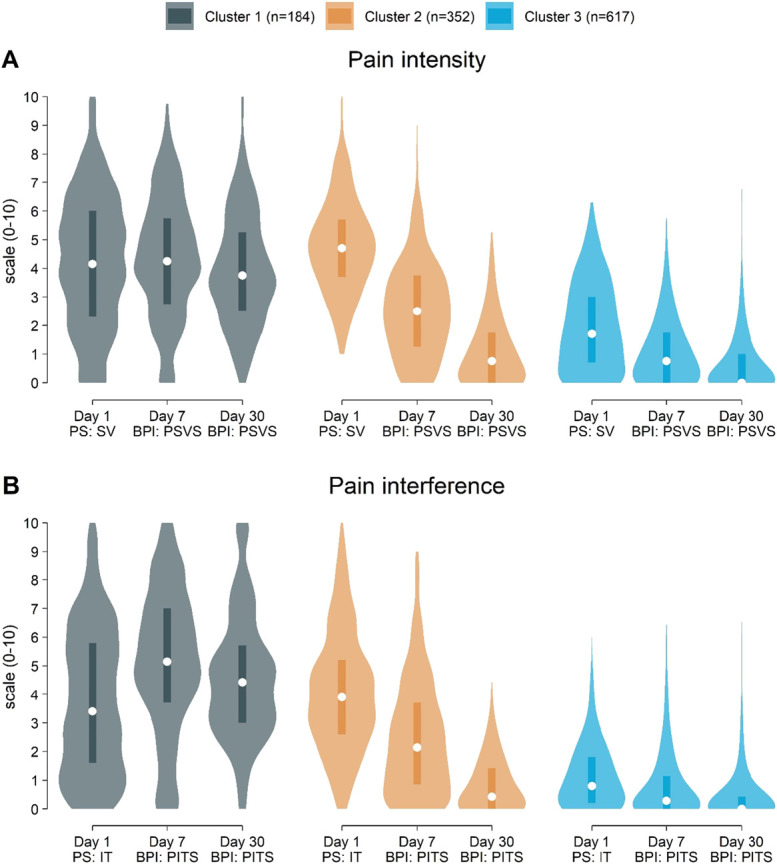
Characterization of the 3 clusters: cluster 1—“Minimal-pain resolution,” cluster 2—“Slow-pain resolution,” cluster 3—“Fast-pain resolution” for day 1, day 7, and day 30 after surgery. (A) Variables for assessing pain intensity: POD1 PS: SV—Pain Severity Score from the International Pain Outcomes Questionnaire; POD7 and 30 BPI: PSVS—Pain Severity Scale of the Brief Pain Inventory. (B) Variables for assessing pain interference: POD1 PS: IT—Pain Interference Score from the International Pain Outcomes Questionnaire, POD7 and 30 BPI: PITS—Pain Interference Scale of the Brief Pain Inventory. The white dots represent the median, and the bar represents the interquartile range. The width of the shaded area (violin plots) corresponds to the frequency of patients with similar values on the *y*-axis (probability density function).

Patient characteristics and perioperative treatments for the patients in clusters 1 to 3 are shown in Table 3 and Figure 1.

**Table 3:** Patient characteristics and process variables for the 3 patient clusters.

	Cluster 1 “**Minimal-pain resolution**” N = 184	Cluster 2 “**Slow-pain resolution**” N = 352	Cluster 3 “**Fast-pain resolution**” N = 617	*P*	ES
Age (y), median (IQR)	46 (34–61)	46 (33–59), n = 351	52 (39–64), n = 615	**<0.001**	0.01 (small)
Duration of surgery (h), median (IQR)	2.2 (1.6–3.5)	2.6 (1.7–3.9), n = 348	2.3 (1.7–3.5), n = 602	0.16	<0.01
Time to survey after surgery (h), median (IQR)	24.3 (20.7–26.0), n = 176	24.4 (19.2–26.2), n = 346	24.3 (20.6–26.1), n = 584	0.64	<0.01
Sex (female), n (%)	149/182 (81.9%)	241/348 (69.3%)	380/612 (62.1%)	**<0.001**	0.15 (small)
Preexisting chronic pain (yes), n (%)	81 (44.0%)	115/351 (32.8%)	139/615 (22.6%)	**<0.001**	0.17 (small)
Opioids taken before admission to hospital	19/181 (10.5%)	24/349 (6.9%)	24/612 (3.9%)	0.0027	0.10 (small)
Surgical discipline (%)				**<0.001**	0.24 (small)
General surgery	46 (25.0%)	156 (44.3%)	236 (38.2%)		
Obstetrics/gynecology	71 (38.6%)	86 (24.4%)	132 (21.4%)		
Thoracic surgery	32 (17.4%)	34 (9.7%)	121 (19.6%)		
Traumatology/orthopedics	31 (16.8%)	48 (13.6%)	55 (8.9%)		
Urology	4 (2.2%)	28 (8.0%)	73 (11.8%)		
Intraoperative anesthesia, n (%)				**<0.001**	0.16 (small)
Regional (only)	64 (34.8%)	99/349 (28.4%)	128/608 (21.1%)		
General (only)	41 (22.3%)	120/349 (34.4%)	253/608 (41.6%)		
Regional and general	79 (42.9%)	130/349 (37.2%)	227/608 (37.3%)		
Intraoperative wound infiltration (yes), n (%)	22/180 (12.2%)	35/341 (10.3%)	100/602 (16.6%)	**0.02**	0.08 (small)
Opioid: intraoperative, n (%)				**<0.001**	0.10 (small)
None	4 (2.2%)	16 (4.5%)	35 (5.7%)		
Systemic (only)	126 (68.5%)	259 (73.6%)	491 (79.6%)		
Regional (only)	32 (17.4%)	46 (13.1%)	53 (8.6%)		
Systemic and regional	22 (12.0%)	31 (8.8%)	38 (6.2%)		
Opioid: recovery room (any), n (%)	46 (25.0%)	107 (30.4%)	206 (33.4%)	0.09	0.06
Opioid: ward (any), n (%)	93 (50.5%)	205 (58.2%)	371 (60.1%)	0.07	0.07
Nonopioid: analgesics intraoperative (any), n (%)	109 (59.2%)	262 (74.4%)	519 (84.1%)	**<0.001**	0.21 (small)
Nonopioid: analgesics recovery room (any), n (%)	32 (17.4%)	68 (19.3%)	161 (26.1%)	**0.009**	0.09 (small)
Nonopioid: analgesics ward (any), n (%)	168 (91.3%)	316 (89.8%)	542 (87.8%)	0.36	0.04

In cases of missing data, the number of patients in the analysis is specified.

In cases with missing data, the number of patients included in the analysis is specified.

ES: effect sizes vary by type of variable, Cohen's ω (categorical variables; absolute values ≥0.1 small, ≥0.3 medium, ≥0.5 large) or eta squared (metric variables; absolute values ≥0.01 small, ≥0.06 medium, ≥0.14 large); IQR, interquartile range; *P*: *P* value of the chi-squared tests (categorical variables) or Kruskal-Wallis tests (metric variables), *P* values <0.05 are written in bold.

Pain and pain-related interference. Compared with patients in clusters 2 (“Slow-pain resolution”) and 3 (“Fast-pain resolution”), those in cluster 1 (“Minimal-pain resolution,” n = 184, 16%) were characterized by higher levels of pain severity and interference on POD1, POD7, and POD30, with little change over time. Patients in cluster 2 (n = 352, 30.5%) also reported relatively high levels of pain severity and interference on POD1, but these decreased gradually by POD30. Finally, patients in cluster 3 (n = 617, 53.5%) reported lower levels of pain severity and interference at all 3 time points.

Acute neuropathic pain (ANeuP) and analgesics. On POD7, patients in cluster 1 (46.2%) reported the highest rate of positive screening for ANeuP compared with patients in cluster 2 (25.3%) and cluster 3 (9.2%). The proportion of patients taking any analgesic did not differ between cluster 1 (92.9%) and cluster 2 (89.5%) but was significantly lower in cluster 3 (74.2%). On POD30, patients in cluster 1 (39.7%) presented the highest rate of screening for ANeuP compared with those in cluster 2 (16.8%) and cluster 3 (8.6%). Patients in cluster 1 took the highest proportion of analgesics (73.2%) compared with 40.5% in cluster 2 and 6.45% in cluster 3.

### 3.3.2. Associative analyses

We found significant differences in patient characteristics and process variables among the 3 clusters (Table 4), which may have prognostic value in identifying cluster 1 membership.

**Table 4:** Patient-reported outcomes and pain treatment for the cluster on postoperative days (PODs) 1, 7, and 30.

	Cluster 1 “**Minimal-pain resolution**” N = 184 (16%)	Cluster 2 “**Slow-pain resolution**” N = 352 (30.5%)	Cluster 3 “**Fast-pain resolution**” N = 617 (53.5%)	1 vs 2		1 vs 3		2 vs 3	
				*P*	ES	*P*	ES	*P*	ES
POD 1									
Pain score: total, median (IQR)	3.2 (1.8–4.8)	3.4 (2.6–4.6)	1.2 (0.6–1.8)	**0.024**	−0.10	**<0.001**	0.46	**<0.001**	0.72
Pain score: severity, median (IQR)	4.2 (2.3–6.0)	4.7 (3.7–5.7)	1.7 (0.7–3.0)	**0.013**	−0.11	**<0.001**	0.42	**<0.001**	0.66
Pain score: interference, median (IQR)	3.4 (1.6–5.8)	3.9 (2.6–5.2)	0.8 (0.2–1.8)	0.076	−0.08	**<0.001**	0.47	**<0.001**	0.68
POD 7									
PSVS: total, median (IQR)	4.3 (2.8–5.8)	2.5 (1.3–3.8)	0.8 (0.0–1.8)	**<0.001**	0.41	**<0.001**	0.59	**<0.001**	0.46
PITS: total, median (IQR)	5.1 (3.7–7.0)	2.1 (0.9–3.7)	0.3 (0.0–1.1)	**<0.001**	0.49	**<0.001**	0.63	**<0.001**	0.47
DN2 (positive), n (%)	85 (46.2%)	89 (25.3%)	57 (9.2%)	**<0.001**	0.21	**<0.001**	0.41	**<0.001**	0.22
Analgesics, n (%)									
Any	170/183 (92.9%)	314/351 (89.5%)	454/612 (74.2%)	0.26	0.06	**<0.001**	0.19	**<0.001**	0.18
Nonopioid analgesics	157/183 (85.8%)	291/351 (82.9%)	430/612 (70.3%)	0.46	0.04	**<0.001**	0.15	**<0.001**	0.14
Opioid	62/183 (33.9%)	119/351 (33.9%)	152/612 (24.8%)	0.99	0.00	**0.02**	0.09	**0.003**	0.10
Co-analgesics	20/183 (10.9%)	26/351 (7.4%)	23/612 (3.8%)	0.23	0.06	**<0.001**	0.13	**0.020**	0.08
POD 30									
PSVS: total, median (IQR)	3.8 (2.5–5.3)	0.8 (0.0–1.8)	0.0 (0.0–1.0)	**<0.001**	0.67	**<0.001**	0.68	**<0.001**	0.24
PITS: total, median (IQR)	4.4 (3.0–5.7)	0.4 (0.0–1.4)	0.0 (0.0–0.4)	**<0.001**	0.76	**<0.001**	0.72	**<0.001**	0.26
DN2 (positive), n (%)	73 (39.7%)	59 (16.8%)	53 (8.6%)	**<0.001**	0.25	**<0.001**	0.36	**<0.001**	0.12
Analgesics, n (%)									
Any	134/183 (73.2%)	142/351 (40.5%)	159/612 (26.0%)	**<0.001**	0.31	**<0.001**	0.41	**<0.001**	0.15
Nonopioid analgesics	114/183 (62.3%)	132/351 (37.6%)	146/612 (23.9%)	**<0.001**	0.24	**<0.001**	0.34	**<0.001**	0.15
Opioids	44/183 (24.0%)	37/351 (10.5%)	39/612 (6.4%)	**<0.001**	0.18	**<0.001**	0.24	**0.029**	0.07
Co-analgesics	24/183 (13.1%)	17/351 (4.8%)	14/612 (2.3%)	**0.001**	0.15	**<0.001**	0.21	**0.049**	0.07

DN2, *Douleur Neuropathique* 2; ES, effect size Cohen's ω (categorical variables; absolute values ≥0.1 small, ≥0.3 moderate, ≥0.5 large) or r (metric variables; absolute values ≥0.1 small, ≥0.3 moderate, ≥0.5 large); IQR, interquartile range; *P*: *P* value of the chi-squared tests (categorical variables) or Mann-Whitney *U* tests (metric variables), *P* values <0.05 are printed in bold; PITS, Pain Interference Scale; PSVS, Pain Severity Scale. The analysis of analgesics specifies the number of patients affected by the missing data. Co-analgesics include anticonvulsants and antidepressants as adjunctive therapies for neuropathic pain.

In the full model (Table 5), a higher risk of cluster 1 membership was associated with patients reporting chronic preexisting pain (vs none) and undergoing thoracic and trauma/orthopaedic procedures (vs general surgery; all *P* < 0.05). In addition, a longer duration of surgery was associated with a higher risk of cluster 1 membership (*P* = 0.014).

**Table 5:** Results of the logistic regression models for cluster 1 membership (vs not; all models N = 1,043 patients).

	Full model		Reduced model*	
	OR (95% CI)	*P*	OR (95% CI)	*P*
Intercept	0.17 (0.07–0.43)	**<0.001**	0.25 (0.14–0.45)	**<0.001**
Surgical discipline (vs general surgery)				
Obstetrics/gynecology	1.48 (0.80–2.72)	0.21	1.66 (0.92–2.99)	0.09
Thoracic surgery	2.16 (1.19–3.90)	**0.011**	2.08 (1.18–3.65)	**0.010**
Traumatology/orthopedics	2.39 (1.28–4.45)	**0.006**	2.71 (1.51–4.81)	**<0.001**
Urology	0.28 (0.07–0.81)	**0.039**	0.27 (0.06–0.79)	**0.036**
Age				
z-score	0.94 (0.78–1.15)	0.56	—	—
Sex (vs female)				
Male	0.59 (0.36–0.94)	**0.029**	0.58 (0.36–0.92)	**0.023**
Preexisting chronic pain (vs no)				
Yes	2.08 (1.42–3.03)	**<0.001**	2.07 (1.43–3.00)	**<0.001**
Duration of surgery				
z-score	1.29 (1.05–1.59)	**0.014**	1.33 (1.09–1.60)	**0.003**
Time to survey after surgery				
z-score	0.97 (0.79–1.19)	0.76	—	—
Intraoperative anesthesia approach (vs general only)				
Regional only	1.58 (0.79–3.12)	0.19	—	—
Regional and general	1.49 (0.92–2.46)	0.11	—	—
Intraoperative wound infiltration (vs no)				
Yes	1.34 (0.74–2.35)	0.32	—	—
Opioid: not systemic administration (vs systemic only)				
None	0.45 (0.12–1.26)	0.16	—	—
Regional (only)	1.02 (0.53–1.96)	0.94	—	—
Systemic and regional	1.28 (0.67–2.37)	0.44	—	—
Opioid: recovery room (vs none)				
Yes	0.91 (0.59–1.40)	0.67	—	—
Opioid: ward (vs none)				
Yes	0.88 (0.58–1.33)	0.55	—	—
Nonopioid: intraoperative (vs none)				
Yes	0.46 (0.27–0.78)	**0.004**	0.40 (0.25–0.65)	**<0.001**
Nonopioid: recovery room (vs none)				
Yes	0.72 (0.44–1.15)	0.18	0.69 (0.43–1.08)	0.11
Nonopioid: ward (vs none)				
Yes	1.07 (0.57–2.12)	0.83	—	—

OR (95% CI), odds ratio including 95% confidence interval (ORs >1 indicate a higher risk for cluster 1 membership, ORs <1 indicate a lower risk for cluster 1 membership), *P*, *P* value of the OR, *P* values <0.05 are printed in bold.

* Variables were backwards removed based on the Akaike Information Criterion.

Patients were at a lower risk of belonging to cluster 1 if they were male (vs female), underwent urological surgery (vs general surgery), and received a nonopioid intraoperatively (vs none; all *P* < 0.05).

The reduced model yielded similar results (Table 5). Nonopioid treatment in the recovery room was associated with a lower risk of cluster 1 membership (*P* ≥ 0.05) in both models.

## 4. Discussion

This observational study evaluated 1,153 patients receiving routine clinical care across 5 surgical disciplines to characterize pain intensity, pain-related functional interference, and early neuropathic pain (ANeuP) during the first month after surgery. When the analysis was performed across the entire cohort, pain and related outcomes improved significantly between POD7 and POD30. By both POD7 and POD30, median scores for pain interference—physical (including sleep) and affective (mood, enjoyment of life, relationships with others)—indicated that pain was either not or only mildly interfering with patients' daily lives. A similar pattern was observed for pain intensity. Use of analgesics, opioid and nonopioid, was much reduced, also indicative of recovery. However, the cluster analysis revealed greater nuance. By POD30, the majority of the cohort, 84% of patients (combining clusters 2 and 3, “Slow- and Fast-pain resolution”), had largely recovered; their scores were indicative of no or mild pain intensity and interference. However, patients in these 2 clusters differed in the rate of recovery from POD1, in the extent that patients screened positive for ANeuP, and in the proportion of analgesics patients were still taking. In contrast, patients belonging to the “Minimal-pain resolution” cluster (cluster 1), forming 16% of the cohort, showed minimal improvement throughout. From POD1 up until POD30, these patients continued to experience mild to moderate pain and interference with function, with 40% screening positive for ANeuP, and 73% were still taking analgesics, including opioids in 24% of patients.

### 4.1. Assessing pain and interference in the acute and subacute phases

Studies do not often assess the association between pain and functional disability in the acute and subacute postoperative periods.^14^ In a single-centre, observational study of patients undergoing orthopaedic surgery, most reported pain in the subacute period (POD10), which interfered with activities of daily living and was associated with pain persisting at 12 months.^37^ The authors suggest that the findings point towards the need for increased follow-up and more intensive management of post-discharge pain for improving quality of life as well as potentially preventing persistent pain. In another single-centre study, poorer pain interference at 2 weeks after orthopaedic surgery was predicted by patients undergoing lower extremity surgery, experiencing worse preoperative pain interference and worse preoperative pain.^14^ Relying on preoperative data limits the clinical practicality of these findings. In a large multicentre study of patients undergoing mixed surgical procedures assessed from POD1–POD7, pain intensity, physical function, and adverse effects improved in parallel, indicating that pain and disability are closely linked early after surgery.^38^ However, responses varied widely in this cohort, highlighting the limits of population-level analyses for understanding postoperative pain patterns and for personalizing care.

### 4.2. Clusters in the acute and subacute phases and what we can learn about risk factors?

In the current study, the cluster analysis was ancillary. However, the findings may be more clinically relevant compared with the sample-based analysis, as we identified distinct postoperative pain subgroups, along with their associated risk and protective factors. Describing patterns of pain-related recovery may expand the options for tailoring personalized care. In this study, we identified a group of high-risk patients. In terms of personalized or individualized medicine, particular attention should be paid to patients in the cluster with no- or Minimal-pain resolution. This applies to acute pain management, but also potentially to more targeted follow-up care after discharge. Early detection and targeted treatment may prevent undertreatment and improve long-term outcomes for these patients. While regression analyses offered preliminary indications for identifying these patients, the preoperative or early postoperative identification of such patients in routine clinical practice remains unsatisfactory at present. The extent to which the integration of preoperative biomarkers (eg, sensitization processes and/or genetic factors^18,30^), psychological variables (eg, pain catastrophizing, anxiety, depression^18,30,34^), intraoperative nociception^27^ and wound size,^34^ or other postoperative variables such as complications (eg, wound infection) and, in particular, early postoperative pain management can improve identification must be investigated in the future. In our cohort, the three-cluster solution demonstrated that higher risk of belonging to cluster 1 (“Minimal-pain resolution”) was linked with orthopaedic/trauma or thoracic surgery, preexisting chronic pain, and longer procedures. A multicentre study identified 3 pain clusters in patients on POD1.^19^ Patients in the cluster with the poorest outcomes (19% of the cohort) were associated with institutional factors, preoperative opioid use, orthopaedic procedures, younger age, higher opioid doses in the recovery room, male sex, and number of comorbidities. Assessing outcomes within 36 hours after oral and maxillofacial surgery revealed that the cohort was divided into 3 clusters, with pain intensity mirroring surgical invasiveness.^32^ Five trajectories were identified in a study assessing diverse surgical procedures across POD1–7, with younger age, female sex, anxiety, and greater pain behaviours increasing the likelihood of high-pain membership.^36^ The authors concluded that trajectories were driven largely by patient rather than surgical factors. Finally, in a study of adolescents undergoing major musculoskeletal surgery, those who reported higher pain and interference scores and a slower recovery trajectory up until POD30 were more likely to develop CPSP 4 months after surgery.^20^ These findings are additional support for the advantage of early monitoring to flag patients at risk. A systematic review of preoperative risk factors for pain within the first 7 days after surgery, evaluated in 48 studies and 23,037 patients, identified 4 significant predictors of postoperative pain intensity: preexisting pain (acute or chronic), anxiety, age (younger), and type of surgery (major orthopaedic, thoracic, and open abdominal procedures). Sex was not a consistent predictor.^13^

### 4.3. Screening for acute neuropathic pain

This study is among the few assessing postoperative ANeuP.^1,11,17,25,31^ Cross-study comparisons are difficult due to methodological differences, surgical populations, sample sizes, and assessment times. Screening for ANeuP is challenging due to the considerable overlap between the symptoms of suspect postoperative ANeuP (eg, burning pain, allodynia, hyperalgesia) vs inflammatory pain, vs primary and secondary hyperalgesia (to touch, cold, heat). The latter is indicative of nociceptive pain and the former of central sensitization.^40^ Nonetheless, studies consistently show that some patients screen positive for NeuP at various postoperative times (POD0, POD2, month 1,^1^ POD2,^17^ POD3,^25^ up to POD6^11,31^), all within the timeframe in which ANeuP is defined. Patients screening positive for ANeuP report higher pain scores in the acute^1,11,17,31^ and chronic^17,25,31^ postsurgical periods. At 12 months after surgery, patients screening positively for neuropathic pain reported worse pain and greater functional interference compared with patients whose pain was not neuropathic.^28^ Early detection of NeuP is relevant as pharmacotherapy recommendations differ for patients with and without NeuP.^6^

### 4.4. Study limitations and strengths

Study limitations include a notable dropout rate between POD1 and POD7, despite up to 3 post-discharge contact attempts, highlighting challenges in patient follow-up. Comparison of demographics, pain outcomes, and treatments between completers and dropouts showed no significant differences in pain-related PROs. Although some characteristics differed, effect sizes were small to trivial, suggesting minimal risk of bias (see supplemental digital content, Material 6, http://links.lww.com/PR9/A415). We developed a digital outcome questionnaire for post-discharge use but found that few patients opted for it, preferring phone contact with a surveyor. Although the DN2 questionnaire is validated for screening neuropathic pain in chronic pain populations, it has yet to be validated in postoperative patients. Availability of simple, symptom-based tools like the DN2 may be useful for screening ANeuP.

Strengths include the substantial mixed-surgery cohort, standardized methodology, and comprehensive assessment of the biopsychosocial dimensions of pain beyond intensity alone. Given the large sample size, we prioritized effect sizes over *P* values to avoid overstating clinically trivial differences.^9^ In addition, it is appropriate to use regression-based analysis to control for confounders in an uncontrolled study design when a randomized controlled trial is impractical. Finally, the cluster analysis uncovered patterns that would have been missed by cohort-level evaluation alone.

## 5. Conclusion

By 1 month after diverse surgeries, most patients in this observational cohort had recovered in terms of both pain and function (physical and affective) when the analysis was carried out at the group level. However, the cluster analysis identified 3 subgroups, so that while pain resolved in the majority of patients, about one-fifth showed minimal improvement and continued to report mild to moderate pain with functional interference. These patients screened more often as positive for ANeuP and still took analgesics, including opioids. Risk factors included preexisting chronic pain and undergoing orthopaedic or thoracic surgery. Identifying at-risk patients preoperatively (based on planned procedure) or early postoperatively (based on nonresolving pain outcomes) may allow for more effective management of pain.

## Disclosures

The authors have no conflict of interest to declare.

## Supplemental digital content

Supplemental digital content associated with this article can be found online at http://links.lww.com/PR9/A415.

**Table:** 

Hospital	Surveyors
Instituto Nacional de Ciencias Medicas y Nutrición Salvador Zubiran (INCMNSZ)	Dra. Lisette Castro Garces Dra. Fernanda Zuleyka Grajeda Rábago Dr. Williams Ramírez Miguel Dra. Andrea Parra Galindo Dr. Pedro Alejandro Saldaña Villaseñor Dr. Hector Olvera Prado Dra. Diana Elizabeth Diaz Arizmendi Dra. Sarahi Sofia Ibañez Barzalobre Dra. Karen Landeros Cruz Dra. Shahaira Jamileth Montejo Romo Dra. Mariana Ornelas Perea Dra. Nabila Cruz Yedra Dr. Anthony Steven Rublee Insignares Dra. Alejandra Garza Villaseñor Dr. Eduardo Antonio Wilson Manriquez
Hospital Central “Dr. Ignacio Morones Prieto”	Dr. Marco Antonio Hernández Govea Dr. Diego Armando Catillo Ramos Dr. Rodrigo Arteaga Alfaro
Instituto Nacional de Cancerología (INCAN)	Dra. María Luisa Sánchez Hernández
Hospital de Especialidades Centro Médico Nacional Siglo XXI, IMSS	Dra. Alma Delia Patiño Toscano
Instituto Nacional de Enfermedades Respiratorias “Ismael Cosío Villegas” (INER)	Dra. María de los Ángeles Macias Jiménez Dra. Susana Gloria Quispe Ticona
Hospital Aranda de la Parra	Dr. Jorge Alejandro Cruz Ponce
Hospital Fundación Médica Sur	Dr. Christopher Bryan Moisen Moreno Dra. María José Martínez Solórzano
Hospital San Javier	Dr. Oscar Lopez Hernandez Dra. Valeria Baltazar Beltran
Hospital General “Dr. Rubén Leñero”	Dr. Miguel Ángel Arce Bernal
Hospital Ángeles Puebla	Dr. Moises Daniel Serrano Merlin
Hospital de Traumatología y Ortopedia Lomas Verdes	Dra. Luna Alonso Yvhonne Dr. Edgar Luis Villegas Esquivel
Hospital General de Villa Coapa	Dra. Paula Imelda Cazares Baraja Dra. Alicia Elena Tamayo Lievanos
